# Effectiveness of Brain Gym Exercises Over Cognitive Behavioural Therapy in Improving Sleep Quality Among Healthcare University Students: A Comparative Study

**DOI:** 10.7759/cureus.58463

**Published:** 2024-04-17

**Authors:** Vaishnavi M Thakre, Mitushi Deshmukh, Joel Gibbs

**Affiliations:** 1 Musculoskeletal Physiotherapy, Ravi Nair Physiotherapy College, Datta Meghe Institute of Higher Education & Research, Wardha, IND; 2 Psychology, Ravi Nair Physiotherapy College, Datta Meghe Institute of Higher Education & Research, Wardha, IND

**Keywords:** health care university, insomnia, pittsburgh sleep quality index, insomnia severity index, cognitive behavioural therapy, physiotherapy, exercises, brain gym

## Abstract

Background

More than half of healthcare university students are believed to experience inadequate sleep. The application of brain gym exercises is a relatively new concept that has shown potential for addressing a variety of cognitive and perceptual deficits. Nevertheless, its use in managing sleep disorders is still in the early stages. Though cognitive behavioral therapy (CBT) is still the standard treatment for sleep management, recent research suggests that brain gym exercises may help with sleep disorders like insomnia. Independent studies have demonstrated the efficacy of CBT and brain gym activities in treating sleep disorders. Yet, there remains a paucity of studies directly comparing these two methods in treating individuals with sleep disturbances.

Aim and objectives

To ascertain the effectiveness of brain gym exercises over cognitive behavioral therapy in improving sleep quality among healthcare university students.

Method

The experimental study comparing brain gym exercises and cognitive behavioral therapy interventions was conducted in Wardha, Maharashtra, India, from March 2023 to December 2023, with 60 subjects selected after considering inclusion and exclusion criteria. The protocol was administered for 4 weeks for 30 minutes per day. The baseline outcome measures were the insomnia severity index (ISI) and the Pittsburgh sleep quality index (PSQI). The outcome measure was used before and after 4 weeks of interventions to evaluate the outcome of the protocol. The software used in the analysis was SPSS 27.0 version and GraphPad Prism 7.0 version, and p<0.05 was considered as the level of significance.

Result

Post-treatment, evidence was observed for ISI (20.55, P = 0.0001) and PSQI (18.09, P = 0.0001) in Group A. Post-rehabilitation evidence was observed in Group B for ISI (19, P=0.0001) and PSQI (16.64, P=0.0001). The findings revealed a significantly higher mean difference between Group A and Group B, showing a considerable improvement in outcome measure scores within Group A relative to Group B.

Conclusion

The statistical analysis demonstrates a notable improvement in the pre-and post-scores of the ISI and PSQI following four weeks of brain gym exercises. In comparison, cognitive behavioral therapy showed comparatively less effectiveness in addressing insomnia. The observed improvement in sleep quality among students highlights that brain gym exercises have the potential to be a non-pharmacological alternative for managing mild to moderate insomnia.

## Introduction

Sleep is ubiquitous in living organisms and fulfills their vital functions [1]. It is a trait of all human entities, takes up one-third of living existence [2], and has been proven crucial for well-being, including mental, physical, and emotional health [3]. Sleep facilitates the body's recovery from activity, maintaining optimal consequential functioning [4]. Sleep is a complex activity involving much more than just closing an individual's eyes. It is a physiologically induced active state of unconsciousness in which the brain enters a state of rest and primarily responds to internal stimuli [5]. Sleep exhibits immune system functioning, lessens caloric consumption, revives brain energy, fulfills a glymphatic function, retains waking-induced performance deterioration, and maintains neuronal and glial connectivity-plasticity [6]. Furthermore, sleep has been proven to enhance recall memory, mediate metabolism, and mitigate mental stress [7]. A sequence of sleep cycles is established through alternating phases of rapid eye movement (REM) (75 to 80%) and non-rapid eye movement (NREM) (20 to 25%) sleep [8]. The initial NREM-REM sleep cycle typically spans 70 to 100 minutes, while subsequent cycles are longer, averaging 90 to 120 minutes [9,10].

Sleep disorders can appear as claims of inadequate sleep, excess perceived sleep, or undesirable movements while sleeping [11]. Poor or inadequate sleep is linked to various abnormalities involving various body systems, including the immune, metabolic, and endocrine systems, jeopardizing higher cortical functions, post-physical task recovery, cognitive performance, and mood [12]. Medical aspirants consist of one subset of the community that appears to be particularly susceptible to sleep deprivation [13]. Challenges with sleep exert a considerable influence on the daily functioning of students. It is estimated that approximately 60% of undergrad students report having inadequate sleep [14], and 7.7% fall under the diagnostic criteria of insomnia [15]. It occurs mainly due to consummate and prolonged study hours, hospital postings encompassing overnight on-call duties, and emotionally taxing work [16-18]. Students go through varying challenges and need to deal with different stressors and evolving situations, affecting their mental well-being and disrupting their sleep quality [19].

Brain Gym is a training program that emphasizes educational kinesiology and neuroscience theories. It was founded in 1987 [20]. It is a form of structured aerobic exercise involving integrated eyes, head, and cross-lateral, balance-necessitating movements that stimulate the left and right brain hemispheres mechanically via the sensory and motor cortexes, revitalizing our natural healing mechanisms [21,22]. It is an incredible personal development tool that allows individuals to render rapid transformations in coping with stress and sleep disturbances, enhancing eyesight, boosting self-esteem, and restoring harmony and health [23,24].

Cognitive behavioral therapy (CBT) is a psychosocial intervention [25] that intends to alleviate symptoms of varied mental health conditions [26], most notably anxiety problems and depression [27,28]. For obtaining sleep improvements, CBT is reported to be more beneficial than standard medical management approaches such as sleep advice and medicines [29,30]. In healthcare universities, more than half of the students are suspected to suffer from poor sleep quality [15]. Brain gym exercises are a recent trend that has successfully treated various cognitive and perceptual impairments. However, its use in treating sleep disturbances is still gaining a stronghold. Recent studies have suggested that brain gym exercises can also be used to reduce sleeping disturbances like insomnia.

In contrast, cognitive behavioral therapy is considered a conventional treatment for managing sleep disturbances [23]. Brain gym exercises and CBT have been proven separately to effectively manage sleep disturbances [24,29]. However, no study has compared these two techniques for treating individuals with sleep disturbances. Thus, our study needed to find the effectiveness of brain gym exercises over CBT in improving sleep quality among healthcare university students with moderate sleep disturbances.

## Materials and methods

The experimental study comparing two interventions was conducted in Wardha, Maharashtra, India, from March 2023 to December 2023. This study was approved by the Institutional Ethics Committee (IEC) of Datta Meghe Institute of Higher Education and Research (DMIHER), deemed to be a university (DU), reference number DMIHER(DU)/IEC/2023/1011. The healthcare university students having moderate sleep difficulty (9-21 score on the insomnia severity index) between the age criteria of 20 and 24 years and those who had signed consent forms were included in the study. Exclusion criteria were already diagnosed depression, ongoing psychological counseling, cognitive damage, and undertaking psychotic drugs.

Data collection tool and technique

**Figure 1:**
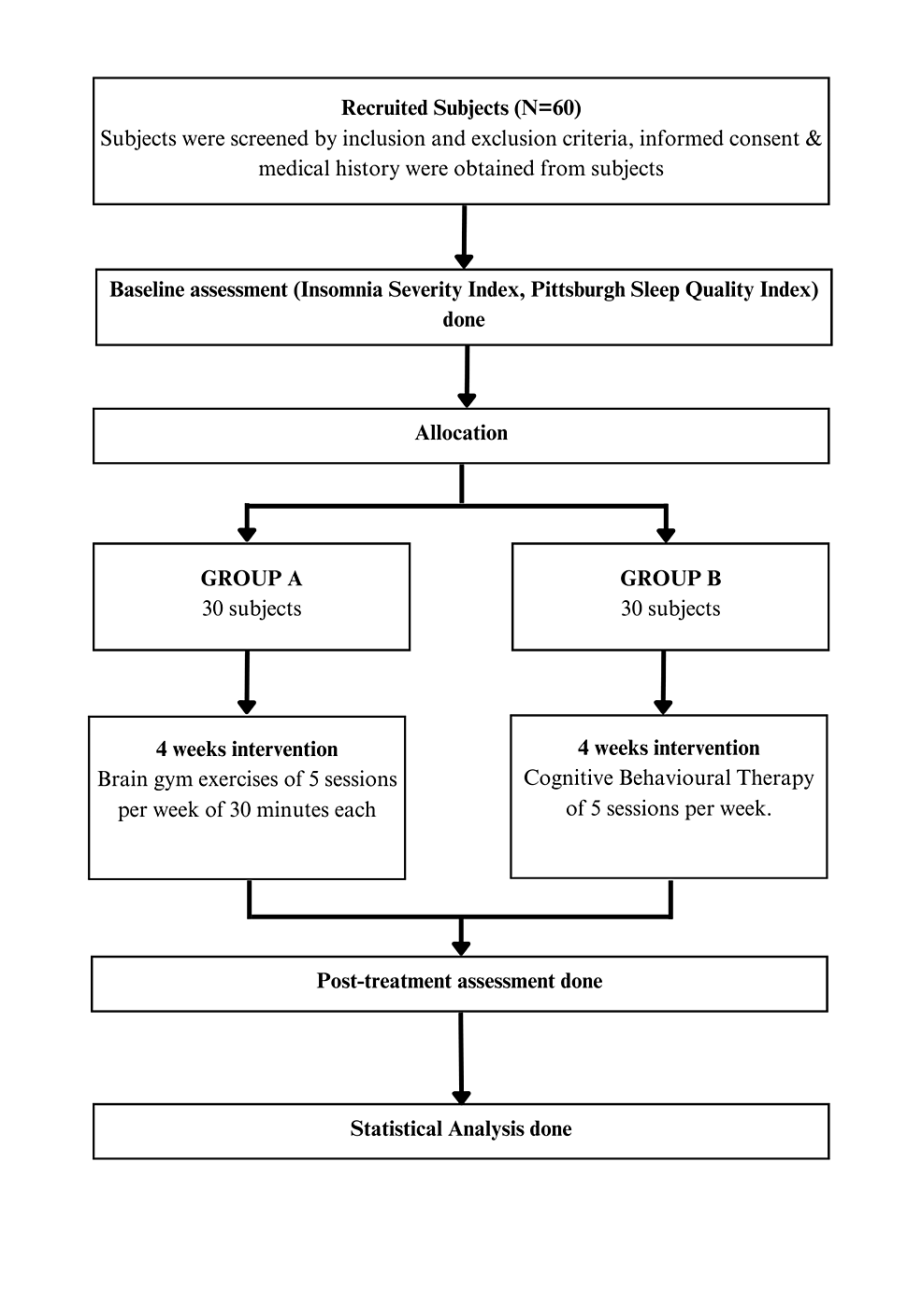
Allocation of samples in two groups

With a sample size of 60 calculated using simple random sampling techniques, the Insomnia Severity Index was used to screen a total of 100 students; of these, 65 were chosen as participants based on the inclusion and exclusion criteria without regard to gender distribution. Before any interventions were administered to the patients who had been chosen, they were extensively examined for vitals. The patients who satisfied the eligibility requirements were included in the study. The study interventions were carried out by a faculty physiotherapist, who assisted the concerned intern. A pre- and post-experimental design was used for the study, where one group received brain gym exercises, and the other group received cognitive behavioral therapy. Patients were separated into Group A (brain gym exercises) and Group B (cognitive behavioral therapy), each with 30 participants. The data collection tool and technique are summarized in Figure 1.

Statistical analysis was done using descriptive and inferential statistics using Student's paired and unpaired t-tests, and the software used in the analysis was SPSS 27.0 version, with p<0.05 considered the level of significance.

Interventions

Group A

They received 30 minutes of brain gym exercises daily, five days per week for four weeks. On the initial day, a demonstration of exercises was given, and later, a home exercise program incorporating Brain gym exercises was prescribed, and an exercise chart was provided for reference. Each brain gym exercise's effects, procedure, and dosage (Table 1).

**Table 1:** Brain gym exercises

	Brain gym exercises	Effects	Procedure	Dosage
1	Spot marching	It primarily serves as a warm-up exercise	The participant assumes an upright position and elevates both legs above the ground in a continuous motion.	One minute
2	Hookups	Promotes relaxation of body and mind	Stand with arms crossed close to your chest, close your eyes, cross your feet, and inhale deeply.	Two minutes (five sets of eight repetitions)
3	Positive points	Reduces stress levels and enhances memory	Closing your eyes, take a deep breath, and press eyeballs gently.	One minute (10 repetitions)
4	The active arms	Activates the brain for tool-controlling skills, relaxation of the diaphragm, and improves hand-eye coordination	Stretch the muscles from the rib cage by extending one arm above and supporting it with the other arm at the elbow. For several seconds, move the extended arm in an isometric manner.	Five minutes
5	Earth buttons	Enhances whole-body orientation and improves mental alertness	With one hand, place two fingers over the lower lip and the other's palm over the navel. Breathe deeply while you look down at the floor, then move your eyes slowly from the floor to the ceiling.	Two minute (10 repetitions)
6	The energy yawn	Facilitates oxygenation	Position the middle and index fingers over the jaw muscles bilaterally, applying gentle pressure while rubbing the muscles in a massaging motion. Open the jaw widely in a prolonged yawning motion, then gently close it.	One minute (10 repetitions)
7	Lazy eights	Boosts concentration, balance, and eye muscle control	Stretch out one arm horizontally in front of the body, forming a figure-eight pattern	One minute (10 repetitions)
8	Gravity glider	Enhances oxygen and blood flow; boosts stability and confidence	Sit comfortably with ankles crossed and knees relaxed, then bend forward while reaching out in front. Allow the arms to glide downward while exhaling, and upward while inhaling. Change the position of the legs and repeat the exercise.	Five minutes (10 repetitions)
9	Foot flex	Promotes socialization and optimise posture; aids in relaxation	Placing the right ankle over the left knee, take a sitting position. Hold the Achilles tendon with one hand while placing the other behind the right knee and grabbing the calf. Repeatedly point and flex the right foot.	Five times for two minutes
10	The energizer	Improves posture; tones back muscles and keeps the spine flexible, supple and relaxed	Position a table in front of you and take a seat on a chair. With your hands on either side of your head, rest your forehead on the table. Lift your head slowly and raise your chin, pointing upward while taking a deep breath. Tuck your chin in and start lowering your head as you exhale. Rest your head on the table for a moment and continue to inhale deeply.	five times for five minutes

**Figure 2:**
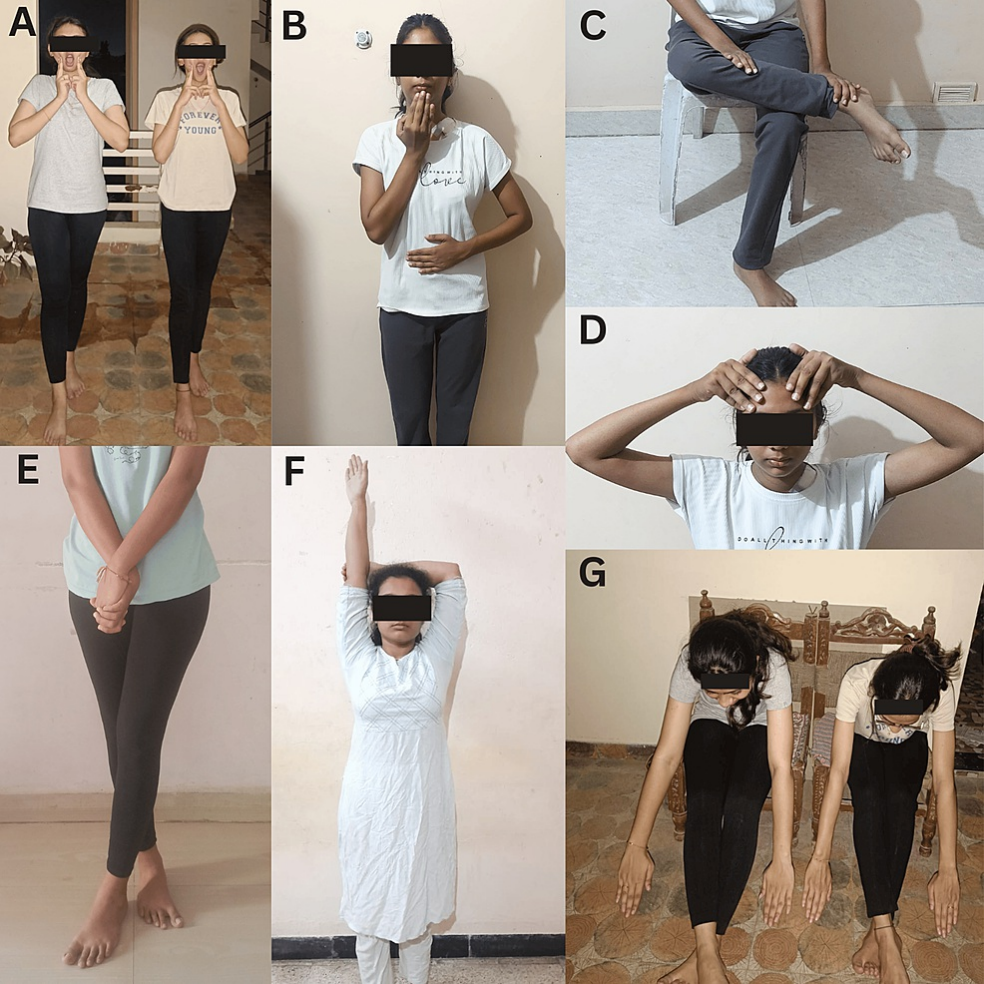
Patients performing brain gym exercises

The patients undergoing spot marching, energy yawns (Figure 2A), earth buttons (Figure 2B), foot flex (Figure 2C), positive points (Figure 2D), hookups (Figure 2E), active arms (Figure 2F), lazy eights, gravity glider (Figure 2G), and energizer.

Group B

They received 1 hour of cognitive behavioral therapy per day, five sessions per week for four weeks under the guidance of a psychologist. After four weeks, a post-treatment evaluation was performed, and data was recorded and calculated using statistical analysis.

Outcome measures

Insomnia Severity Index

It is a highly reliable and valid scale with 86.1% sensitivity and 87.7% specificity for detecting insomnia. A total score of 0-7 indicates no clinically significant insomnia, 8-14 means subthreshold insomnia, 15-21 means clinical insomnia (moderate severity), and 22-28 means clinical insomnia (severe) [31].

Pittsburgh Sleep Quality Index

The Pittsburgh Sleep Quality Index (PSQI) is a self-report questionnaire with 86.5% specificity and 89.6% sensitivity that evaluates sleep quality and irregularities across one month. 18 There are 7 "component" scores obtained by 19 individual items: subjective sleep quality, sleep latency, sleep duration, habitual sleep efficiency, sleep disturbances, use of sleeping medication, and daytime dysfunction. The sum of the scores for these 7 components results in a single global score. The 19 self-rated items are added to obtain seven "component" scores, each ranging from 0 to 3 points. A score of "0" signifies no difficulty, whereas a score of "3" signifies severe difficulty in any setting [32].

## Results

Throughout the study, all registered participants underwent a comprehensive four-week treatment protocol outlined in the research methodology. The baseline characteristics of the participants, encompassing various demographic variables, were thoroughly documented and summarized (Table 2). Statistical analysis of these baseline characteristics found no significant differences between the groups, indicating a well-matched cohort for the study.

**Table 2:** Distribution of patients in two groups according to their age in years SD: Standard deviation; Yrs: years; CBT: Cognitive behavioral therapy

Age Group(years)	Brain Gym Exercise	CBT
17-20 yrs	16 (53.33%)	16 (53.33%)
21-24 yrs	14 (46.67%)	14 (46.67%)
Mean±SD	20.33±1.80	20.80±2.09
Range	18-23 yrs	18-24 yrs

In the brain gym exercise group, the mean insomnia severity index score decreased significantly from 12.80±3.78 on the pre-test to 5.96±2.49 on the post-test, with a mean difference of 6.83±1.82. The t-value obtained from the student's paired t-test was 20.55, which indicates a highly significant difference (p = 0.0001, S). In the CBT group, the mean score was reduced from 12.36±3.75 at the pre-test to 9.20±3.52 at the post-test, resulting in a mean difference of 3.16±0.91. The t-value from the student's paired t-test was 19, indicating a moderately significant difference (p = 0.0001, S). When comparing the two groups using the student's unpaired t-test, the t-value was 0.44 with a p-value of 0.65 (NS), indicating that there was no significant difference between the groups at baseline. However, at the post-test, the t-value increased to 4.09 with a p-value of 0.0001 (S), indicating a significant difference in the outcomes between the brain gym exercise and CBT groups. This showed that Group A showed an immense improvement in scores on the outcome measures compared to Group B. The above results are summarised (Table 3).

**Table 3:** Comparison of Insomnia Severity Index in two groups at pre- and post-treatment S: Significant; NS: Not significant; CBT: Cognitive behavioral therapy

Group	Pre-Test	Post-Test	Mean Difference	Student’s paired t-test p-value
Brain gym exercise	12.80±3.78	5.96±2.49	6.83±1.82	20.55 P=0.0001,S
CBT	12.36±3.75	9.20±3.52	3.16±0.91	19 P=0.0001,S
Comparison between two groups(Student’s unpaired t-test)				
t-value	0.44	4.09	-	-
p-value	0.65, NS	0.0001, S	-	-

The brain gym exercise group's mean score of the PSQI decreased significantly from 10.20±2.77 at the pre-test to 4.50±1.52 at the post-test, with a mean difference of 5.70±1.72. The t-value for the Student's paired t-test was 18.09, suggesting a highly significant difference (p=0.0001, S). In the CBT group, the mean score reduced from 10.53±2.77 at the pre-test to 6.70±2.38 at the post-test, resulting in a 3.83±1.26 mean difference. The Student's paired t-test was 16.64, suggesting a moderately significant difference (p=0.0001, S). When comparing the two groups at post-test using the Student's unpaired t-test, the t-value increased to 4.26 with a p-value of 0.0001 (S), indicating a significant difference in the outcomes between the Brain Gym Exercise and CBT. The above results are summarised (Table 4).

**Table 4:** Comparison of Pittsburgh Sleep Quality Index in two groups at pre and post-treatment S, Significant; NS, Not significant; CBT: Cognitive behavioral therapy

Group	Pre-Test	Post-Test	Mean Difference	Student’s paired t-test p-value
Brain Gym Exercise	10.20±2.77	4.50±1.52	5.70±1.72	18.09 P=0.0001, S
CBT	10.53±2.77	6.70±2.38	3.83±1.26	16.64 P=0.0001, S
Comparison between two groups(Student’s unpaired t-test)				
t-value	0.46	4.26	-	
p-value	0.64, NS	0.0001, S	-	

## Discussion

Sleep accounts for approximately one-third of human existence, although its precise purpose or function remains incompletely understood from a scientific perspective. However, recent research findings suggest that sleep exerts a much greater impact on the brain than previously assumed [2]. Medical students bear a significant academic burden, potentially exacerbating poor sleep quality beyond that observed in the general population. According to the comprehensive worldwide literature review focusing on the sleep experiences of medical students, we observe that inadequate sleep is not only common among this group but also more prevalent compared to non-medical students and the general population [13].

The research aimed to examine the effectiveness of brain gym exercises and CBT in improving sleep quality among healthcare university students. Considering the importance of addressing sleep disturbances through non-pharmacological interventions, the study investigated the potential of brain gym exercises as a viable solution. The beneficial effects of brain gym exercises on improving memory, attention, promoting relaxation and cognitive functions have been the focus of numerous research projects. The PSQI and the Insomnia Severity Index were used to evaluate the results. The ISI is a valid and reliable tool for detecting cases of insomnia in the general population, according to Morin et al. Additionally, they reported that the ISI is sensitive to treatment outcomes among insomnia patients. [31]. In the Brain Gym Exercise group, the mean score decreased significantly from 12.80±3.78 at the pre-test to 5.96±2.49 at the post-test, with a mean difference of 6.83±1.82 while in the CBT group, the mean score was reduced from 12.36±3.75 at the pre-test to 9.20±3.52 at the post-test, resulting in a mean difference of 3.16±0.91. In individuals with primary insomnia, a 6-point reduction in the ISI score is considered a clinically significant improvement [32]. In accordance with Manzar et al., the PSQI, in its original English edition, was deemed beneficial for diagnosing sleep disorders in young adult male Indian university students [33]. After comparing pre and post-PSQI scores, the Brain Gym Exercise group exhibited a mean difference of 5.70±1.72. Meanwhile, the CBT group showed a mean difference of 3.83±1.26. In comparison, a change greater than 4.4 indicates that this change is clinically meaningful [34]. The study revealed clinically significant improvements in sleep quality across both outcome measures within the brain gym exercise group. Evidence ranging from low to moderate quality suggests that cognitive behavioral therapy (CBT) demonstrates superior efficacy compared to medications in treating insomnia, with benefits persisting for six months or longer following the completion of therapy [35].

Brain Gym is a curriculum rooted in neuroscience and educational kinesiology principles. Utilizing the "brain-body link" theory, these exercises help activate the brain to render neurons stronger to deal with an array of external data and respond to a "corporate member" of their duty corresponding to certain brain areas. Brain Gym provides opportunities for personal growth, fostering rapid transformations and enhancing quality of life across various age demographics [22]. After eight weeks of intervention, brain gym substantially alleviates anxiety and poor sleep quality in older adults, as reported by Effendy et al. [36]. The demand for non-pharmacological interventions is on the rise primarily because young individuals who are dealing with stressful lifestyles are more likely to experience difficulties with sleep. The research carried out by Seth et al. underscores the need to extend the application of brain gym exercises to broader age groups, as their findings indicate positive outcomes. This expansion holds the potential for enhancing sleep quality and augmenting memory, cognition, and attention, thereby presenting a viable option for addressing mild to moderate insomnia [37]. According to Kulkarni et al., young adults who engage in a brain gym training program report improving their attention span [23].

The study evaluated the comparative effectiveness of brain gym exercises and cognitive behavioral therapy in managing moderate insomnia among students. Both treatments demonstrated enhanced outcomes; however, patients undergoing brain gym exercises exhibited significant improvements. It investigated the strategies employed to induce relaxation and enhance sleep quality in this population.

Strength and clinical implications

The findings of this study have several implications for the field of physiotherapy and healthcare. Firstly, the study provides evidence for the effectiveness of brain gym exercises in treating patients with sleep disturbances. This is useful for physiotherapists who are treating patients with these conditions. Secondly, the study provides insights into the impact of sleep disturbances on mental health. It contributes to the growing body of research on the topic and may lead to developing new strategies for preventing and treating sleep disturbances in various disorders. Finally, the study provides valuable information for individuals who are suffering from sleep disturbances and its impacts.

Limitations

The sample size was relatively small, and a larger sample size should be used to obtain a more comprehensive analysis. Additionally, the study was conducted over a brief period. A longer-term study could be contemplated to acquire more comprehensive and structured data. Moreover, the study did not encompass factors such as stress measurement tools and specific categories of degree, which should be included in future research.

## Conclusions

Based on the research findings and subsequent discussions assessing the effectiveness of brain gym exercises compared to cognitive behavioral therapy in enhancing sleep quality among healthcare university students, it is evident that brain gym interventions led to a significant decrease in PSQI scores, indicating improved sleep quality. In contrast, cognitive behavioral therapy demonstrated less effectiveness in this regard. The observed improvement in sleep quality among students highlights that brain gym exercises have the potential to be a non-pharmacological alternative for managing mild to moderate insomnia.
